# *A*ngiotensin Converting Enzyme Inhibitors *C*ombined with *E*xercise for Hypertensive *S*eniors (The ACES Trial): Study Protocol of a Randomized Controlled Trial

**DOI:** 10.3389/fmed.2019.00327

**Published:** 2020-01-22

**Authors:** Sara A. Harper, Liliana C. Baptista, Lisa M. Roberts, Sarah J. Wherry, Rebecca S. Boxer, Kerry L. Hildreth, Regina S. Seay, P. Hunter Allman, Christy S. Carter, Inmaculada Aban, Wendy M. Kohrt, Thomas W. Buford

**Affiliations:** ^1^Division of Gerontology, Geriatrics and Palliative Care, Department of Medicine, University of Alabama at Birmingham, Birmingham, AL, United States; ^2^Center for Exercise Medicine, University of Alabama at Birmingham, Birmingham, AL, United States; ^3^Integrative Center for Aging Research, University of Alabama at Birmingham, Birmingham, AL, United States; ^4^Division of Geriatric Medicine, Department of Medicine, University of Colorado Anschutz Medical Campus, Denver, CO, United States; ^5^Kaiser Permanente Colorado, Institute for Health Research, Aurora, CO, United States; ^6^Department of Biostatistics, University of Alabama at Birmingham, Birmingham, AL, United States

**Keywords:** exercise, aging, functional status, antihypertensive, hypertension

## Abstract

Prior evidence suggests that the choice of antihypertensive medication may influence functional status among older adults with hypertension, particularly in conjunction with exercise. In particular, angiotensin converting enzyme (ACE) inhibitors have shown potential to positively influence function. However, randomized, controlled trials are needed to confirm this hypothesis. This paper outlines an RCT designed to determine if choice of first-line antihypertensive medication influences functional and cardiovascular risk factor responses to exercise among older adults with hypertension. Two hundred and thirteen inactive, community-dwelling adults ≥60 years of age with hypertension and functional limitations will be recruited to engage in a 32-week intervention study. Participants will be randomized to one of three first-line antihypertensive agents: (1) the ACE inhibitor perindopril, (2) the AT1 receptor antagonist losartan, or (3) the thiazide diuretic hydrochlorothiazide (HCTZ). Six weeks after randomization, participants will begin a 20-week structured aerobic exercise intervention. Participants will perform two 45-min center-based sessions coupled with 60 min of home-based walking per week. The primary aim is to determine if perindopril improves self-paced gait speed when compared with losartan and HCTZ. The secondary aim is to determine the relative effect of perindopril on secondary outcomes such as: (a) exercise capacity, (b) body mass and composition, and (c) circulating indices of cardiovascular risk. This RCT is expected to identify differential effects of first-line antihypertensive medications when combined with physical exercise thus have potential implications for antihypertensive prescription guidelines for older adults.

**Clinical Trial Registration:** www.ClinicalTrials.gov, identifier: NCT03295734.

## Introduction

The loss of physical function in advanced age is associated with not only the onset of disability and the loss of independence, but also increased rates of cardiovascular morbidity and mortality (1–3). For instance, declines in self-paced walking speed are associated with increased risk of stroke (4), adverse outcomes following cardiac surgery (5), and all-cause mortality (1, 3, 6). Compared to normotensive counterparts, older persons with hypertension experience accelerated declines in walking speed (7, 8), and increased rates of disability (9, 10). Thus, interventions are needed to preserve function and attenuate risk of associated adverse events among hypertensive older adults.

Currently, physical exercise is commonly considered the standard intervention for improving physical function among older adults (11–14). However, the extent of functional benefits from exercise are variable and extensive evidence suggest that antihypertensive medications—particularly those which mediate the renin-angiotensin system (RAS) may influence functional outcomes (15, 16). Moreover, there is inconsistency in the literature regarding the impact of specific antihypertensive drug classes. To address potential differences in antihypertensive drugs, three commonly prescribed medications were chosen based on the following criteria: (1) the ability to improve physical function, (2) tested in similar trials acting with different but complementary biological mechanisms, (3) demonstrated benefits in improving physical performance, (4) considered innovative for affecting mobility outcomes, (5) safety records, and (6) broadly available at low cost. Thus, an angiotensin converting enzyme (ACE) inhibitor, Perindopril, was selected due to potential superiority compared to other ACE inhibitors for preventing cardiovascular outcomes (17) and improving physical function (18). For comparison, AT1 receptor blocker, Losartan that also modulates the RAS inhibiting ligand binding to the angiotensin type 1 receptor, and a diuretic, hydrochlorothiazide (HCTZ) that does not modulate the RAS system (19). While conflicting data do exist, pre-clinical and clinical evidence from our group (20–25) suggest that, among first-line antihypertensive medications, Angiotensin Converting Enzyme (ACE) inhibitors may promote the greatest functional responses to exercise. The potential beneficial effects are associated with pleiotropic effects in the regulation of oxidative stress, inflammation, and angiogenesis-related adaptations to skeletal muscle that may be independent of lowering blood pressure (26–28).

As a first step toward testing this hypothesis, we previously conducted a pilot randomized control trial (RCT) to refine the study protocol and to assess the safety and feasibility of study interventions in the target population (29, 30). This study demonstrated that the study protocol was safe and generally feasible while identifying specific challenges which must be overcome to conduct a fully-powered trial. The current manuscript reflects the lessons learned from this pilot study and outlines the RCT designed to determine if choice of first-line antihypertensive medication influences functional and cardiovascular risk factor responses to exercise among older adults with hypertension. The primary aim is to determine if, compared to the AT1 receptor antagonist losartan and the thiazide diuretic hydrochlorothiazide (HCTZ), the ACE inhibitor perindopril improves self-paced gait speed. The secondary aim is to determine the relative effect of perindopril on secondary outcomes such as: (a) exercise capacity, (b) body mass and composition, and (c) circulating indices of cardiovascular risk. This RCT is expected to identify differential effects of first-line antihypertensive medications when combined with physical exercise and thus have potential implications for antihypertensive prescription guidelines for millions of older adults with hypertension.

## Study Design/Methods

### Overview

The ACE Inhibitors Combined with Exercise for Hypertensive Seniors (ACES) trial is a multi-site, triple-masked RCT of community-dwelling older adults with hypertension and functional limitations. The trial has been approved by the University of Alabama at Birmingham Institutional Review Board (#000000637). A total of 213 participants will be recruited and randomized to treatment groups for the study. Eligible participants will be randomized to receive one of either (1) perindopril, (2) losartan, or (3) HCTZ for blood pressure management and followed for a total of 32 weeks. Due to safety and practical implications for real-world implementation, the study design will accommodate stratified randomization to account for a prior history of hypersensitivity to ACE inhibitors—commonly evidenced by a pronounced cough (i.e., “cough history”). For those with cough history, they will only be randomized to either ARB or HCTZ. Additionally, randomization will also be stratified by age (60–69, 70+ yr) and site.

**Figure 1:**
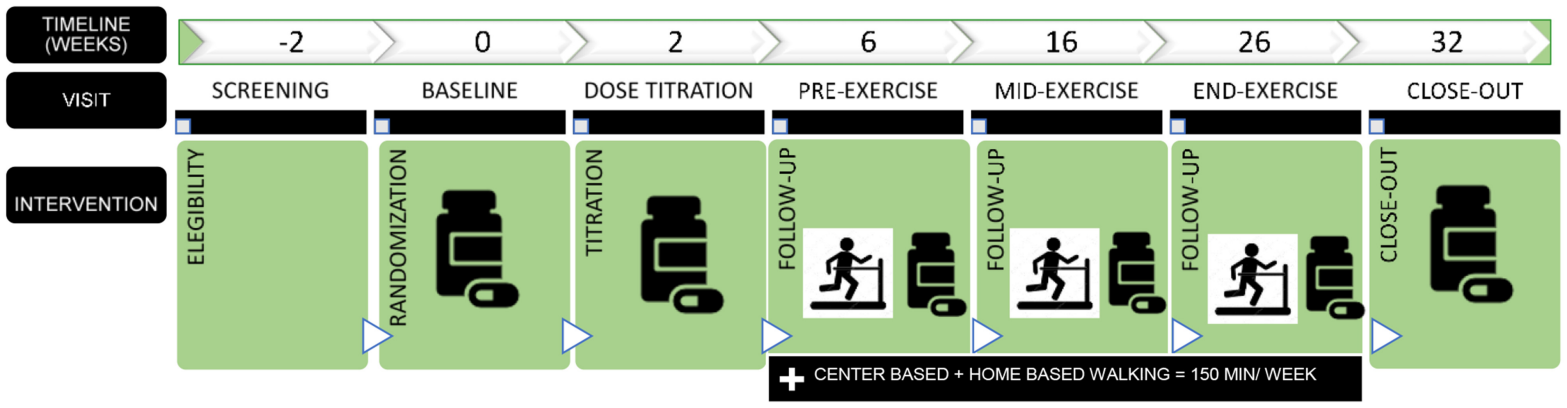
Design overview. Participants will be randomized to one of three first-line antihypertensive medications: (a) perindopril (titration from 4 to 8 mg/day); (b) losartan (titration from 50 to 100 mg/day). If participants do not tolerate the higher study dose will remain in the lower dose of the study medication. (c) HCTZ (from 12.5 to 25 mg/day).

In addition to the randomization to medication, all groups will undergo a 20-week structured center- and home- based aerobic exercise intervention preceded and followed by a 6-week non-exercise period. The initial pre-exercise period will provide a “wash-out” period for prior study medications, while the post-exercise period will provide information regarding the sustainability of effects following cessation of center-based exercise. Following completion of exercise, participants will be provided guidance on continuing exercise in line with public health guidelines. During the 32-week trial, participants will be evaluated for changes in functional limitations and cardiovascular risk outcomes. Study assessments will be conducted at weeks 0, 2, 6, 16, 26, and 32 post-randomization (Figure 1).

To preserve treatment allocation, the randomization and the study medication dispensation will be conducted by an independent university investigational drug service (i.e., research pharmacy). Participant safety will be overseen by a comprehensive study team—including the principal investigator, study clinicians, study staff, and an appointed Data Safety Monitoring Board. The study was designed initially with two recruitment sites (Birmingham, AL and Denver, CO, USA). All participants will provide written informed consent prior to enrollment approved by the Institutional Review Board of the respective participating institution. The study was registered at www.clinicaltrials.gov (NCT03295734) prior to participant enrollment.

### Participants

Two hundred and thirteen community-dwelling persons ≥60 years old with hypertension and objective signs of functional limitations will be recruited from multiple recruitment sites via diverse recruitment approaches including direct mailings, media advertisements (e.g., newspaper, magazine, and radio), and other community approaches. Participant inclusion and exclusion criteria are presented in Table 1. Participants are remunerated for their time and effort required.

**Table 1:** Inclusion and Exclusion criteria for the ACES trial.

**Inclusion criteria**
Age ◾ ≥60 years old
Hypertension- untreated (SBP ≥ 130 mmHg or DBP ≥80 mmHg) or treated
Inactive Lifestyle
◾ <150 min/week of moderate activity on Community Health Activities Model Program for Senior questionnaire (31)
Functional Limitation
◾ >290 s on long-distance corridor test (32)
Willingness to participate in all study procedures
**Exclusion criteria**
Failure to provide consent
Blood pressure >140/90 mmHg despite use of three or more antihypertensive drugs
SBP > 180 mmHg or DBP > 110 mmHg
History of hyponatremia with the use of HCTZ
Chronic kidney disease
Serum creatinine > 2.5 mg/dL in men or 2.0 mg/dL in women
Serum potassium exceeding laboratory reference range
Urinary protein (Albumin) > 1+ on dipstick
Abnormal liver enzymes
◾ AST, ALT, or alkaline phosphatase > 2.5 times the upper limit
Severe cardiac disease including NYHA Class III–IV HF, clinically significant aortic stenosis, history of cardiac arrest, use of cardiac defibrillator, or uncontrolled angina
Subject or objective indicators of ischemic heart disease without follow-up
Significant cognitive impairment (known diagnosis of dementia or Mini-Cog score <3)
Current participation in another intervention trial
Other condition or concern that would preclude participation ◾ Individuals taking beta-blocker at screening for the management of hypertension will be excluded from the trial. If prescribed for another reason, e.g., arrhythmia, the participant may be eligible for the trial if the study clinician and the participant's Primary Care Physician feel it would be safe for the participant to come off the medication for the duration of the trial

*ACE, Angiotensin converting enzyme; ALT, Alanine aminotransferase; AST, Aspartate aminotransferase; HF, Heart failure; DBP, Diastolic blood pressure; HCTZ, Hydrochlorothiazide; SBP, Systolic blood pressure*.

### Screening and Randomization

Interested individuals first participate in a telephone pre-screening interview to discuss the study and assess potential eligibility. Those remaining interested and eligible are then scheduled for an in-person screening visit. At the screening visit, participants sign the informed consent form and are assessed for study eligibility.

**Figure 2:**
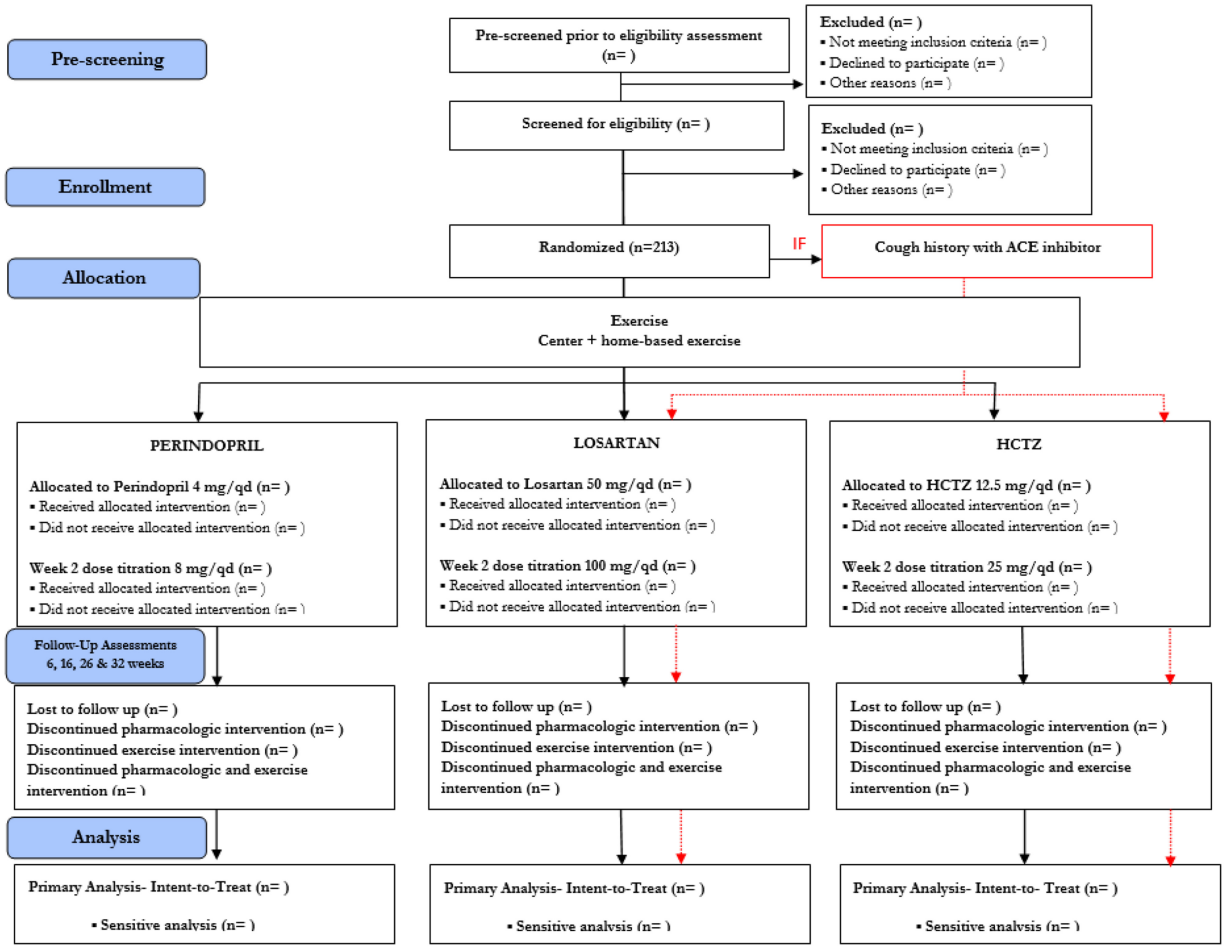
Study Design in Consolidated Standards of Reporting Trials (CONSORT) Format. Due to safety, the study design will accommodate stratified randomization by cough history. Participants with cough history will only be randomized to either Losartan or HCTZ. For those without cough history, they will be randomized to Perindopril, Losartan, and HCTZ.

Participants who meet all eligibility criteria are asked to return for a baseline assessment visit and are randomized (Figure 2). Block randomization, stratified by site (UAB and Colorado) and age (60–69, 70 yr), is used to assign participants to pharmacological intervention arms, with 1:1:1 allocation ratio, to ensure approximately equal accrual to each intervention group throughout the study. Participants with a known hypersensitivity to ACE inhibitors (e.g., cough) are assigned 1:1 to the other two medications. Randomization schedule was created and is maintained by the study biostatistician (IA).

To preserve staff masking, the randomization scheme is performed through a website supervised by the study biostatistician. Study coordinators enter the appropriate randomization stratification scheme [age stratification (60–69, 70+), known adverse events or hypersensitivity to ACE inhibitors, and site location) and participant identification directly to the academic investigational pharmacies that perform the randomization. Treatment allocation remains concealed to all directly involved (investigators, study staff, and participants) until the end of the trial.

### Intervention

#### Pharmacologic Intervention

Eligible participants are randomly assigned to one of three, federal drug administration-approved antihypertensive medications. Notably, although less commonly used in practice in the U.S., the perindopril intervention was chosen based on the existing literature regarding the relative efficacy of various available ACE inhibitors (18, 33, 34). The perindopril intervention starts with a 4 mg/day dose and will be titrated to 8 mg/day after approximately 2 weeks, to safely control participants' blood pressure. The same scheme will also be used with losartan (titration from 50 to 100 mg/day), and HCTZ (from 12.5 to 25 mg/day). If participants do not tolerate the higher study dose due to issues such as hypotension, cough, or hyperkalemia, participants will remain on the lower dose of the study medication. Study medication doses will be adjusted and supervised by the study clinician to control blood pressure. In addition, amlodipine (2.5, 5, or 10 mg) is available as a supplemental drug for participants in all study groups should additional blood pressure control be needed. Potassium chloride may also be prescribed as needed for hypokalemia. To assure double-masking for study staff and participants, study medication will be over-encapsulated with identical capsules (Clinical Encapsulation Services, NY, USA). Study medication will be dispensed at weeks 0, 2, and 16 and returned at weeks 2, 16, and 32 to monitor adherence via pill counts. Any unused study medication will be returned to study staff for adherence records.

#### Exercise Intervention

In addition to the pharmacological intervention, all participants will engage in a 20-week structured exercise intervention beginning at week 6. The intervention will include twice weekly, center-based, aerobic exercise as well as home-based walking. Each center-based session will include 45 min of aerobic exercise in addition to balance and flexibility exercises to promote cool-down. Participants will also engage in an additional 60 min/week of moderate-intensity aerobic exercise outside of center-based session (two-to-three bouts of 20–30 min). This intervention achieves 150 min of aerobic activity per week, meeting clinical and public health guidelines for older persons and those with hypertension (35, 36).

**Figure 3:**
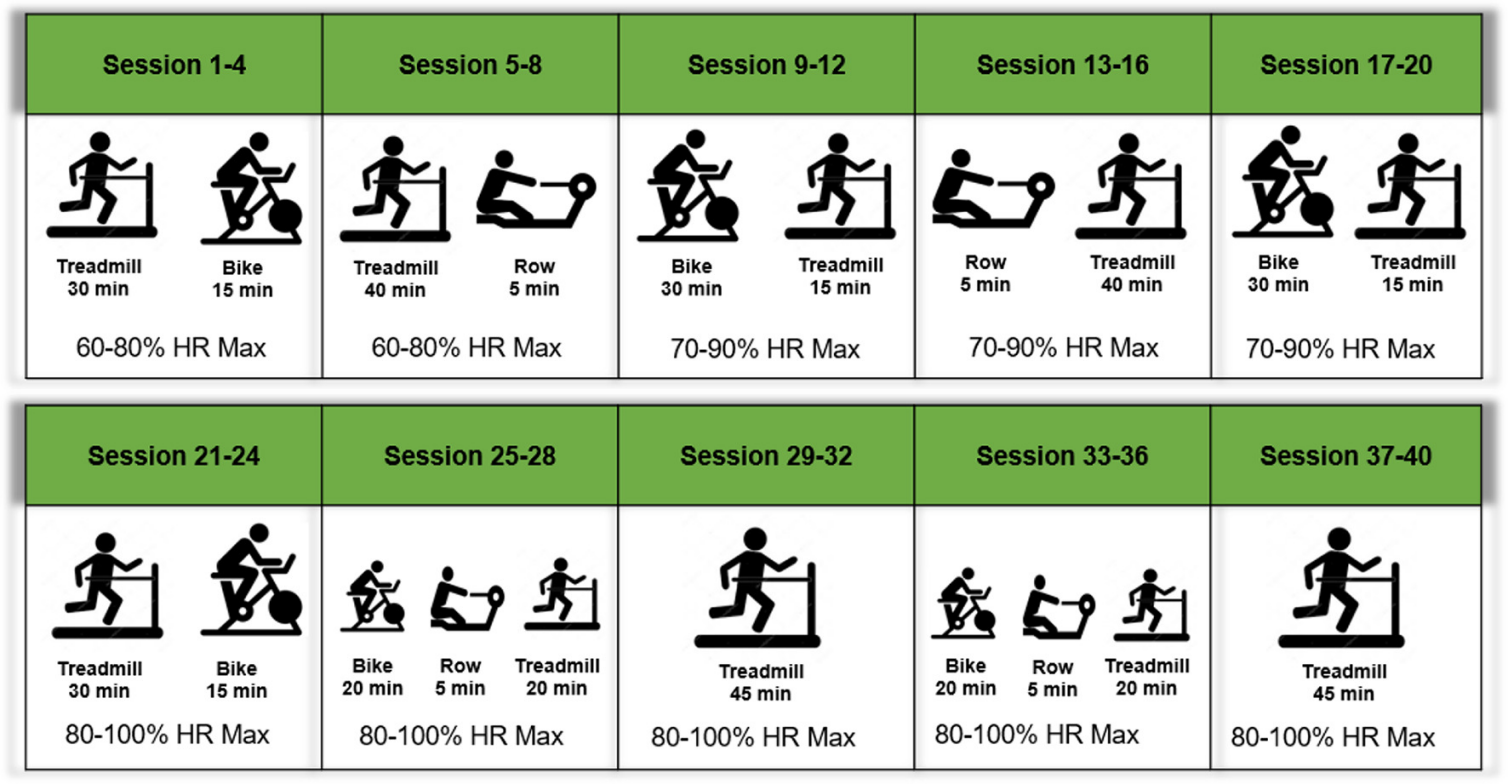
Overview of the exercise intervention.

Center-based sessions begin with a brief warm-up followed by 45 min of moderate-intensity aerobic exercise. Treadmill walking represents the primary form of exercise, supplemented by stationary cycling and stationary rowing to provide variety (Figure 3). Participants will be introduced to the intervention exercises in a structured way such that they begin with lighter intensity and gradually increase during the first weeks of the intervention. In each session, participants will be encouraged to reach a desired exercise intensity range within the minimal and maximal intensity progression block. Participants will be encouraged to maintain the desired heart rate range in each session, with frequent monitoring and encouragement by interventionists. Moreover, progression will be based on sessions attended, rather than week within the trial. Thus, participants must attend four sessions (sessions 1–4) prior to moving for next progression block (Figure 3). Exercise intensity will be monitored with a heart rate monitor (Polar, Lake Success, NY, USA) during exercise sessions.

Home-based walking exercise will be performed at a moderate intensity throughout the intervention [~5–6 Category Ratio 10 scale (37)] and participants will self-report home-based wear time and walking in a written log. In addition, home-physical activity will be monitored objectively via a physical activity tracker FitBit® Zip (San Francisco, CA, USA) throughout the trial providing a measure of time spent in activity, daily steps, estimated caloric expenditure, and distance walked per day. Objective activity data will be monitored by exercise interventionists to provide feedback, encourage engagement, and address barriers when necessary.

### Adherence of Interventions

Adherence of both the pharmacologic and exercise interventions will be monitored throughout the study. The adherence to the pharmacologic intervention will be assessed through pill counting when participants return previously dispensed study medications at weeks 2, 16, and 32. Exercise adherence will be recorded as attendance at each exercise session as well as mean heart rate for each session. The home-based walking exercise will be tracked weekly through the written self-reported intervention logs as well as the objective physical activity monitor.

### Assessment Measures

The primary outcome of interest for the study will be the change in usual-pace walking speed, measured over a 4 m course. Secondary outcomes include exercise capacity, total body fat mass, and fat-free mass, as well as circulating indices of cardiovascular risk. Extended details on the definition and measurement of primary and secondary outcomes are provided below. A timetable of events is also provided (Table 2).

**Table 2:** Data collection summary via assessment visit.

**Study Phase**	**Pre-randomization**		**Pre-exercise**		**Exercise**		**Post-exercise**
**Visit description (FU, follow-up; CO, close-out)**	**Screen**	**Baseline**	**Dose titration**	**FU**	**FU**	**FU**	**CO**
Visit number	1	2	3	4	5	6	7
Week number	−2	0	2	6	16	26	32
Informed consent, review inclusion/exclusion criteria	x						
Personal interview, medical history, medication use	x						
CHAMPS questionnaire	x						
Long corridor walk test	x						
Office blood pressure (sitting + standing), vital signs	x	x	x	x	x	x	x
Anthropometry, ECG, physical exam	x						
Functional measures for screening	x						
Blood and urine for safety labs	x		x	x	x	x	
Randomization		x					
4 m walk, 6-min walk, blood for study assays		x		x		x	x
DEXA, short physical performance battery		x				x	
Dispense study medications		x	x		x		
Collect home BP monitor data			x	x	x	x	x
Charlson comorbidity index		x					
WOMAC pain subscale		x					
Health-related quality of life		x					x
Quick food scan		x		x		x	x
Assess adverse experiences			x	x	x	x	x
Exploratory outcomes		x				x	

*CHAMPS, Community Healthy Activities Model Program for Seniors; CO, Close out; DEXA, Dual energy x-ray absorptiometry; ECG, Electrocardiogram; FU, Follow-up; WOMAC, Western Ontario and McMaster Universities Osteoarthritis Index*.

#### Primary Outcome

Gait speed will be assessed via a 4 m walk test (32). This simple and cost-effective physical performance screening tool will be used to assess functional status (5, 6). Participants will be asked to line up with both feet touching the starting line. After a specific verbal command, the participant will walk a 4 m course at their usual pace. The time needed to complete the entire 4 m course will be recorded. The test is performed twice, with the faster walk used as the outcome for 4 m usual-paced gait speed.

#### Secondary Outcomes

Exercise capacity will be evaluated using the fast-paced six-min walk test (38) as previously described (29). The 6-min walk test is a safe and reliable test of aerobic endurance in older adults (39). Further, the test has a strong reproducibility and a modest correlation with peak oxygen uptake (VO_2_) (1). Briefly, participants will be asked to walk as far and fast as possible for 6 min on a 40 m course. At the end of the test, total distance walked will be recorded.

Body composition will be assessed using dual energy x-ray absorptiometry before and at the end of the exercise intervention (week 26) to determine fat mass and fat-free mass (Lunar iDXA; General Electric Healthcare, Boston, MA, USA and Hologic Discovery W, Marlborough, MA, USA). Body composition analysis will be performed in both total and lower body compartments.

Circulating indices of cardiovascular risk will be assessed through fasted blood samples collected at study visits to assess blood lipids, metabolic, and glucose profiles. Additionally, blood samples will be assayed for inflammation and oxidative stress biomarkers including tumor necrosis factor α (TNF-α), interleukin-6 (IL-6), vascular cell adhesion molecule-1 (VCAM-1), endothelium selectin (E-selectin), oxidized low density lipoprotein (oxLDL), and myeloperoxidase (MPO) using commercially-available ELISA kits.

#### Supportive Measures

Additional supportive outcome measures will be evaluated to aid in the interpretation of study outcomes. The Short Physical Performance Battery (SPPB) will be performed as an additional measure of functional status (40). The SPPB has a strong association between physical function and cardiovascular risk among older adults (1, 39, 41, 42). The Quick Food Scan, a food frequency questionnaire focusing on fruit and vegetable intake (43, 44), will be used to assess dietary intake. The Charlson Comorbidity Index, a method of predicting mortality by classifying or weighting comorbid conditions (comorbidities) (45), will be used to evaluate co-morbidity. The Western Ontario and McMaster Universities Osteoarthritis Index (WOMAC) pain sub-scale will be used to evaluate lower-extremity pain at study entry. Finally, the Short Form 12 (SF-12) a generic health-related quality of life questionnaire (QOL) validated for adults with hypertension (46), will be used to monitor disease burden and to identify patients' perceptions of their health-related QOL.

#### Exploratory Outcomes

We will also collect data on several exploratory outcomes from a voluntary subset of participants. Interested participants will have the option to participate in additional assessments to evaluate (1) changes in VO_2_peak, (2) lower extremity skeletal muscle function, and (3) skeletal muscle indices of angiogenesis. VO_2_peak will be assessed via a graded treadmill exercise test with indirect calorimetry.

To ensure safety of participants, a study clinician will be present for all graded treadmill tests. Participants will be instructed to warm-up on the treadmill at a speed which elicits a minimum of 70% of age-predicted maximum heart rate. During the test, the selected speed stays consistent while the incline grade will increase 2% every 2 min until volitional exhaustion or when the proctor stops the test. A valid VO_2_peak test has been reached when two of the following occur: a plateau in VO_2_ despite an increase in energy demand (change of <0.1 L/min in last three consecutive 20 s averages) or a change in VO_2_ (ml/kg/min) of <2 ml/kg/min over an increasing workload, a respiratory exchange ratio >1.05 or greater, or a maximal heart rate within 10 beats of the age-predicted value.

Skeletal muscle function will be assessed via unilateral isokinetic strength of knee extensors of the dominant limb via a dynamometer as previously published (47, 48). Indices of angiogenesis will be evaluated via the collection and analysis of percutaneous skeletal muscle biopsies. Muscle biopsies will be obtained by a trained, licensed clinician approximately midway between the patella and iliac crest in the vastus lateralis as previously published (49–51). Target outcomes include the (1) proportion of type I (oxidative) muscle fibers, (2) capillaries per muscle fiber, and (3) muscle content of mRNA and proteins related to angiogenesis. Other physiologic pathways will be explored pending availability of tissue.

### Safety

Numerous safety procedures will be utilized to ensure participant safety. First, the exclusion criteria are designed to exclude those with significant risk in participating. In addition, throughout the study, participants will be instructed to record home rested blood pressure three times/day (morning, afternoon, and evening) with an automatic digital home blood pressure monitor (52) (Omron Bp786n 10 series upper arm blood pressure monitor; Horikawa, Japan). Participants will be instructed to report any SBP > 180 mmHg or DBP > 100 mmHg to study staff immediately. Those with two or more elevated blood pressure readings will be scheduled for a clinic visit, at which time the clinician will determine the appropriate course of action for participant safety. Blood pressure will also be monitored at each assessment visit. Participants may be discontinued from the study medication for any home weekly average DBP > 100 mmHg or SBP > 180 mmHg to maintain participant safety. Blood pressure will also be monitored before and after each exercise session. Participants with blood pressure readings of either >180 SBP or >100 DBP at an exercise session will be prohibited from exercise in that day. The clinician may decide to exclude participants with slightly lower blood pressure readings than these cut points if there is any medically-relevant concern that would preclude continued participation. Participants who discontinue study medication will be encouraged to continue the exercise intervention and to perform the assessment visit, in line with an intent to treat approach.

Several clinical chemistry indicators such as creatinine, sodium, potassium, and urinary albumin will be monitored throughout the study. The clinician will have full discretion to manage the prescription of oral potassium chloride for any potassium <3.5 mEq/L. Throughout the intervention, abnormal serum potassium levels will be rechecked every 2 weeks and potassium doses will be increased as needed, until circulating levels are normalized. In addition, the study clinician will adjust study dose appropriately in other adverse clinical responses such as hyperkalemia or hyponatremia. Throughout the intervention, serious and non-serious adverse events will be monitored at each assessment visit and reported. Exercise interventionists will also monitor adverse events as they are reported as well as any potential events that occur during performance of the exercise intervention. If a safety concern arises during the exercise intervention, participants will be discontinued from the intervention and will be encourage to continue with the other study procedures. Finally, participants who are discontinued from the study medication and exercise intervention will be encouraged to complete the assessment visits.

### Sample Size Calculation

The sample size was calculated based upon an anticipated mean difference between groups of 0.055 m/s for 4 m gait speed based upon data from a prior pilot study with a similar population (23). Based on an observed standard deviation of 0.11 m/sec, this value corresponds to a modest effect size of 0.5 which is reasonable for shorter-term (i.e., < 1 year) studies. Moreover, prior literature (53, 54) has demonstrated that this effect size is clinically significant (established range = 0.05–0.10 m/s). Thus, assuming a 80% power, a 13% attrition rate, and a two-sided significance level of 0.05, a total of 213 participants (71 participants per group) are required (calculated via SAS statistical software in line with analytic plan in section 2.10). Additionally, we will have 80% power to detect an effect size ≥0.60 (Cohen's d) for testing the proposed hypotheses on secondary outcomes.

### Statistical Analyses

The primary outcome for the study will be change in 4 m gait speed from week 6 to 26 (the exercise period) in the trial. Prior to all analyses, the assumption of normality will be checked using histograms and normal probability plots. For gait speed, we will use linear mixed effects models with random intercept (55, 56) to account for the temporal correlation among the multiple observations for each subject. Follow-up model-based contrast tests will be applied to test the effect of perindopril compared to losartan and HCTZ.

For all the secondary outcomes other than body mass and composition, we will fit similar linear mixed effects models as for the primary outcome. For the body mass and composition measures, we will use the analysis of covariance (ANCOVA) method. Exploratory outcomes will also be evaluated using ANCOVA. Corrections for multiple comparisons will be utilized within (e.g., four serum inflammatory analytes) but not between (i.e., exercise capacity vs. body composition vs. serum analytes) secondary and exploratory outcomes.

For all outcomes, the primary analysis will include data from all randomized participants, in line with intent-to-treat principles. A secondary analysis will be performed to evaluate changes during the full intervention period (weeks 0–32). For each analysis, sensitivity analyses will be performed to evaluate the impacts of relevant potential confounders including age, sex, race, site, retention (i.e., drop-out), adherence (i.e., medication and exercise). Given that those with cough history cannot be randomized to ACE, additional sensitivity analyses will be performed on just those without cough history to examine the robustness of the primary and secondary analyses based on all randomized patients.

## Discussion

The decline of physical function is an important indicator of independence loss, subsequent disability, and increased risk of cardiovascular-related events. In fact, the decline in self-paced gait speed, a recommended measure of physical performance (57, 58) has been associated with the incidence of stroke and adverse events after cardiac surgery (4), as well as with cardiovascular and all-cause mortality (1, 3, 6). Thus, it is critical to identify interventions addressing the decline of physical function in older adults to preserve functional independence, and decrease the associated cardiovascular-related burden.

Notably, evidence from large clinical trials demonstrated that hypertension is associated with accelerated rate of decline in self-paced gait speed among older adults. In fact, when compared to normotensive counterparts, older adults with hypertension experience accelerated declines in gait speed and higher rates of disability (7, 8, 10, 23). As a result, older adults with hypertension represent a clinically-relevant risk group for functional decline.

To date, physical exercise is widely considered the standard intervention to improve functional status among older adults (59, 60). However, despite the general benefits of physical exercise, these benefits are not universal and the extent of improvement among individuals is quite variable (61, 62). Thus, exercise appears to be a necessary component of treatment regimens to prevent age-related loss of physical function, but further refinement in the prescription is necessary (63).

As previously outlined (29, 30), the choice of first-line antihypertensive medications may have an important role on functional responses after exercise. For instance, ACE inhibitors have been suggested as a promising therapeutic option to target the rate of functional decline, potentially due to pleiotropic effects on body composition, angiogenesis, inflammation and oxidative stress (64, 65). In fact, previous evidence has suggested that ACE inhibitors and exercise combined may attenuate declines in gait speed and on limitations of activities of daily-life (15, 20, 34). Yet, conflicting evidence exists. For instance, Sumukadas et al. (66) reported that ACE inhibitors did not significantly influence functional outcomes among older adults. Given this conflicting evidence, equipoise still exists for the research question requiring further follow-up. Notably, however, the current prior study differs from the present one in several ways including that: (1) the prior study included a mix of hypertensive and non-hypertensive individuals, (2) did not directly compare ACE inhibitors to other approved antihypertensive treatments, and (3) the study intervention period included only 10 weeks of supervised exercise which included functional and strength-based exercises, but not aerobic training. Given these differences and the lack of consistency in the literature, questions remain making the present study an important contribution to knowledge in this area.

In summary, this trial is expected to differentiate the effects of three first-line antihypertensive medications on functional status and cardiovascular risk factors among older adults with hypertension. This study design was informed by several successes and challenges encountered as lessons learned during the pilot study phase, as described previously (30). Briefly, changes from the pilot phase to facilitate success include: (1) the implementation of the study in a larger metropolitan area and use more sites to increase recruitment numbers, (2) focus on aerobic exercise only, (3) pre- and post-exercise intervention periods for wash-out and follow-up for sustainability of effect, and (4) use an objective device to measure home physical activity to prevent the subjectivity of self-reported instruments and participant's burden. Ultimately, this project has potential implications for influencing clinical practice guidelines in the prescription of antihypertensive drugs to millions of older adults.

## Author Contributions

TB: conceptualization and supervision and project administration. TB, WK, CC, and IA: methodology. TB and WK: investigation. IA, PA, TB, SH, LB, and LR: data curation. SH, LB, LR, and TB: writing—original draft preparation. All authors: writing—review and editing. TB, WK, and CC: funding acquisition.

### Conflict of Interest

The authors declare that the research was conducted in the absence of any commercial or financial relationships that could be construed as a potential conflict of interest.
